# The Effect of Visual Experience on Perceived Haptic Verticality When Tilted in the Roll Plane

**DOI:** 10.3389/fnins.2017.00687

**Published:** 2017-12-06

**Authors:** Luigi F. Cuturi, Monica Gori

**Affiliations:** Unit for Visually Impaired People, Science and Technology for Children and Adults, Istituto Italiano di Tecnologia, Genoa, Italy

**Keywords:** perception, haptic perception, verticality, blindness, visual impairment, vestibular function tests, proprioception

## Abstract

The orientation of the body in space can influence perception of verticality leading sometimes to biases consistent with priors peaked at the most common head and body orientation, that is upright. In this study, we investigate haptic perception of verticality in sighted individuals and early and late blind adults when tilted counterclockwise in the roll plane. Participants were asked to perform a stimulus orientation discrimination task with their body tilted to their left ear side 90° relative to gravity. Stimuli were presented by using a motorized haptic bar. In order to test whether different reference frames relative to the head influenced perception of verticality, we varied the position of the stimulus on the body longitudinal axis. Depending on the stimulus position sighted participants tended to have biases away or toward their body tilt. Visually impaired individuals instead show a different pattern of verticality estimations. A bias toward head and body tilt (i.e., Aubert effect) was observed in late blind individuals. Interestingly, no strong biases were observed in early blind individuals. Overall, these results posit visual sensory information to be fundamental in influencing the haptic readout of proprioceptive and vestibular information about body orientation relative to gravity. The acquisition of an idiotropic vector signaling the upright might take place through vision during development. Regarding early blind individuals, independent spatial navigation experience likely enhanced by echolocation behavior might have a role in such acquisition. In the case of participants with late onset blindness, early experience of vision might lead them to anchor their visually acquired priors to the haptic modality with no disambiguation between head and body references as observed in sighted individuals (Fraser et al., 2015). With our study, we aim to investigate haptic perception of gravity direction in unusual body tilts when vision is absent due to visual impairment. Insofar, our findings throw light on the influence of proprioceptive/vestibular sensory information on haptic perceived verticality in blind individuals showing how this phenomenon is affected by visual experience.

## Introduction

Perceiving the direction of gravity, i.e., verticality, is of great importance in building references that are used to maintain balance and move through space. Perception of verticality is often involved in clinical application aimed to aid diagnosis of brain strokes (for a review: Pérennou et al., 2014) and vestibular deficits (Brandt et al., 1994; Böhmer and Rickenmann, 1995; Bisdorff et al., 1996). Several studies have investigated perception of verticality using psychophysical methods focusing on the subjective visual vertical (SVV; for a review: Mittelstaedt, 1983), subjective haptic vertical (SHV; Tarnutzer et al., 2012; Fraser et al., 2015) and the subjective auditory vertical (Harris and Carnevale, 2015) leading to interesting findings especially related to the influence of vestibular and proprioceptive sensory information on the estimation of the direction of gravity. Already in 1861, Aubert discovered a systematic bias in estimating verticality when tilted on a side (Aubert, 1861): the so called A-effect shows that in these conditions, estimation of verticality is tilted toward body tilt. This phenomenon has often been interpreted as an expression of body tilt underestimation due to the influence of an idiotropic vector signaling the upright position (Mittelstaedt, 1983; De Vrijer et al., 2008, 2009) that in Bayesian terms could be modeled as a prior set at the most common body orientation relative to gravity, that is 0° on the roll plane (MacNeilage et al., 2007). Functional perception of verticality is important in order to maintain postural stability as it signals the direction of gravity when standing upright. Deviations from this orientation need to be signaled to avoid unwanted falls; in this sense, biases when tilted could be considered as a byproduct of an efficient system that improves precision in graviception when standing upright. The effect opposite to the A-effect is called E-effect (where “E” stands for Entgegengesetzt, that is “opposite” in German) and it appears when verticality estimations are away from body tilt (Müller, 1916). Further studies observed that perceived verticality switches from A- to E-effects when the tilt overcomes 135–150° (Kaptein and Van Gisbergen, 2004; Kaptein et al., 2005) and this effect is related to a change in precision that likely depends on the effectiveness of the otolith organs in transducing head orientation relative to gravity (Tarnutzer et al., 2009).

However, the origin of such biases is still controversial. Regarding the influence of either vestibular or proprioceptive sensory information on the readout of verticality, studies involving neurological patients show a strong influence of the somatosensory rather than vestibular system in inducing an A-effect (Bronstein, 1999). Moreover, manipulation of the body center of mass influences the haptic subjective vertical showing biases that likely follow the muscular compensation in response to the changed center of mass on the spinal axis (Fourre et al., 2009). Interestingly, this effect is not registered when the center of mass is modified only at the head level (Fourre et al., 2009). At the same time, galvanic stimulation acting on vestibular afferents from the otoliths and the semicircular canals affects judgments of verticality during (Mars et al., 2001) and after stimulation (Volkening et al., 2014) suggesting a strong role given by vestibular sensory information in encoding perception of verticality. Considering the encoding sensory modality (e.g., visual or haptic), the scenario is also quite diverse. Although in the case of the subjective visual vertical (SVV), most studies have found strong A-effects when the whole body is tilted (Ceyte et al., 2009) and when only the head relative to the body is tilted (Fraser et al., 2015), some studies observed E-effects especially for small tilts (Clemens et al., 2011). On the other hand, results relative to the subjective haptic vertical (SHV) are more controversial as some studies found biases toward the body tilt (Bortolami et al., 2006; Fraser et al., 2015), others away from the body tilt (Bauermeister et al., 1964; Schuler et al., 2010) and some others no strong biases in any direction (probably driven by high interindividual variability; Tarnutzer et al., 2012). However, Fraser et al. (2015) have observed that verticality judgments based on haptic cues access mostly body coordinates whereas visual judgments are mostly performed within head coordinates; this view supports the model of Clemens and colleagues that poses verticality as based on an internal estimate of gravity simultaneously accessed by the brain within head or body reference frames (Clemens et al., 2011).

Generally, the importance of perception of gravity is observed in behaviors related to spatial navigation and falls avoidance. These tasks can be critical when faced by individuals characterized by severe visual impairments, such as blindness. Although compensatory theories support the idea that the lack of vision results in improvements in perception through the remaining senses, this is not always the case regarding spatial navigation (Seemungal et al., 2007; for a review: Cuturi et al., 2016) and regarding the integration of other sensory cues in order to accurately move through space (Nardini et al., 2008; Gori et al., 2017). This view is also supported by the importance of vision during the first stages of development in guiding multisensory integration underlying perception of objects orientation (Gori et al., 2008). Nevertheless, early experience of vision does not seem to influence accuracy in perceiving oblique orientations whereas there are differences between sighted and blind people (Gentaz and Hatwell, 1998). On the other hand, many blind people take advantage of the echo produced by a self-emitted sound in order to aid successful orientation in space, that is echolocation (for a review: Kolarik et al., 2014).

Blindness is a unique condition to evaluate whether visual experience influences perception of gravity with regards to the vestibular and proprioceptive sensory information. Nevertheless, no previous studies have investigated whether the absence of vision either from birth or later in development influences perception of verticality when tilted in a non-upright orientation. Our aim in this study is to investigate this topic by using on orientation discrimination task with subjects tilted 90° on the left-ear side. Additionally, we tested whether the position of the stimulus relative to the head on the body longitudinal axis has an effect on the perceived verticality and how this aspect is influenced by blindness onset and echolocation behavior. Representation of surrounding space can differ depending on the stimulus position relative to the body accessing different coordinates (Soechting et al., 1990). Our interest here is to test whether this aspect might change depending on the visual experience and the sensory systems available. To this aim, we tested three experimental conditions where subjects were tilted and had to judge the orientation of a plastic bar presented in three different positions on the longitudinal body axis: aligned with the head, above the head or below the head. This was done in order to disclose whether head, peripersonal (i.e., above the head position) and body (i.e., below the head position) reference frames had a role in the perceived verticality when tilted on a side. Considering also the findings of Fraser and colleagues on differences in perceived verticality depending on the tilt of the head relative to the body and vice versa (Fraser et al., 2015), we expect that different positions of the stimulus on the longitudinal axis might unveil the access to different reference frames and possibly influencing perceived verticality when tilted. Moreover, visual experience might have a role in influencing the access to different coordinates systems depending on the sensory modality in use (Pouget et al., 2002). In order to test for inherent biases in judging bar's orientation when standing upright, we ran a baseline condition where subjects judged the horizontality instead of verticality in order to keep the same bar orientation relative to the body. Our results show that there are no biases in the baseline condition while we observe biases when subjects are tilted. In particular, visually impaired individuals show different estimations depending on the blindness onset and their echolocation behavior during daily life thus suggesting that an idiotropic vector useful for spatial navigation may develop thanks to early experience of vision and echolocation.

## Materials and methods

### Participants

Forty-seven human subjects participated in this experiment (30 sighted subjects, mean age = 27 y.o., SD = 8, 14 female, all except one subject right handed; 17 visually impaired subjects, mean age = 43 y.o., SD = 15, 6 female): 34 completed the baseline condition, 43 completed the below-head condition, 39 completed the head condition and 35 completed the above-head condition. Details about visual impairment and participants characteristics are shown in Table 1. All participants provided signed informed consent before starting the test. This study was approved by the ethics committee of the local health service (Comitato Etico, ASL 3, Genova, Italy).

**Table 1:** Characteristics of visually impaired participants.

	**Age at test**	**Gender**	**Pathology**	**Onset of blindness**	**Echolocation behavior**	**Baseline**	**Head**	**Above-head**	**Below-head**
**EARLY BLIND**									
EB_01	27	F	Retinopathy of Prematurity	Birth	Yes	X	X	X	X
EB_02	28	F	Retinitis pigmentosa	Birth	No	[ ]	X	[ ]	[ ]
EB_03	78	F	Retinitis pigmentosa	Birth	Yes	X	X	X	X
EB_04	25	M	Leber's amaurosis	Birth	No	X	X	X	X
EB_05	51	M	Retinopathy of Prematurity	Birth	Yes	X	X	X	X
EB_06	57	M	Uveitis	Birth	No	X	X	X	X
EB_07	58	M	Congenital glaucoma	Birth	No	X	X	X	X
**LATE BLIND**									
LB_01	51	F	Leber's amaurosis	46	No	X	X	X	X
LB_02	26	F	Optical nerve tumor	6	No	X	X	X	X
LB_03	38	F	Retinitis pigmentosa	30	No	[ ]	X	X	X
LB_04	49	M	Retinitis pigmentosa	40	No	X	X	X	X
LB_05	29	M	Corneal opacity	17	Yes	X	X	X	X
LB_06	26	M	Leber's amaurosis	13	Yes	X	X	X	X
LB_07	45	M	Congenital glaucoma	6	No	X	X	X	X
LB_08	54	M	Optic chiasm tumor	14	No	X	X	X	X

*The table represents age at the time of testing, gender, pathology, age (y.o) at which subjects became completely blind, self-report of performing echolocation in daily life and participation in each experimental procedure (X indicates participation)*.

### Stimuli

**Figure 1:**
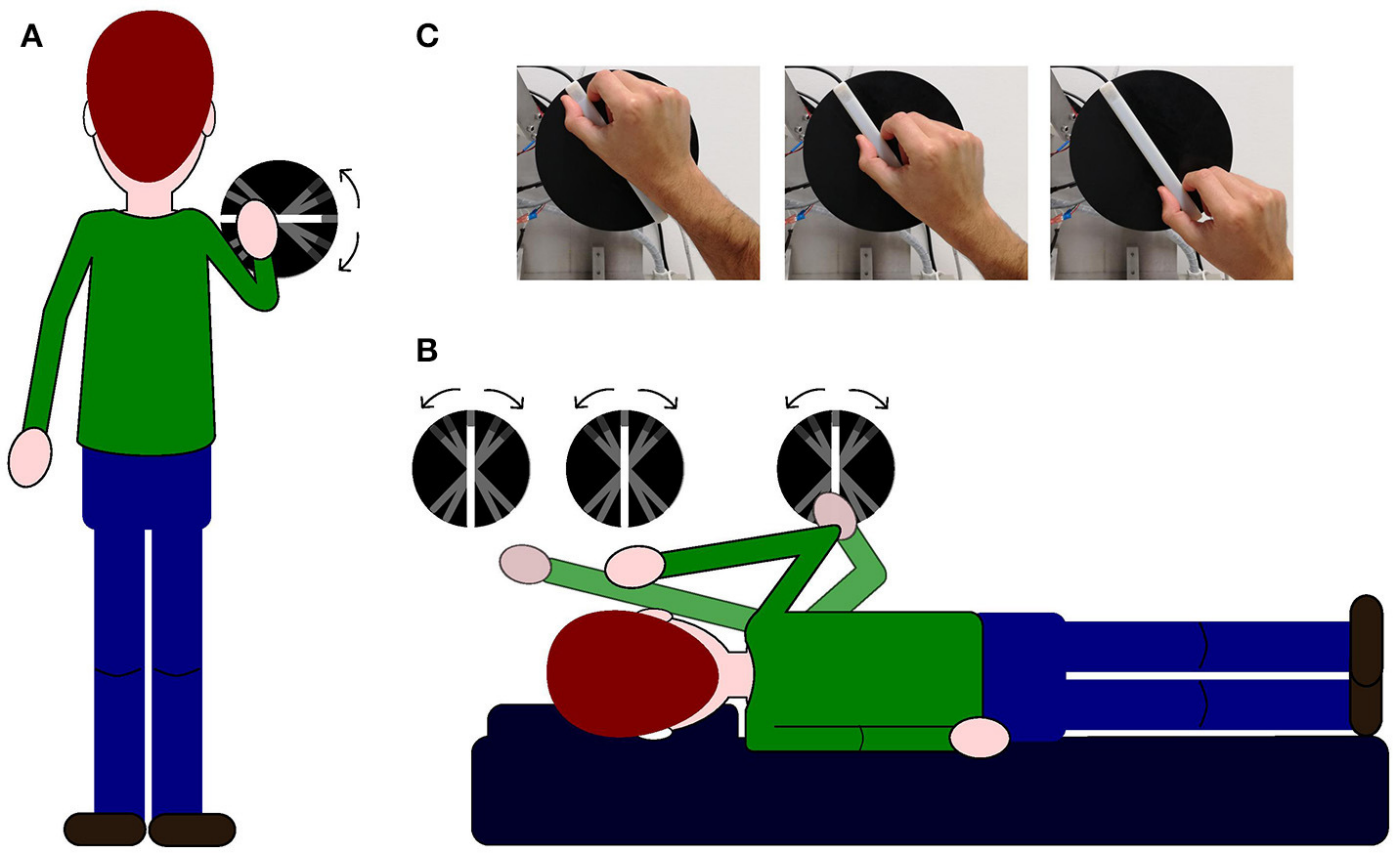
Illustrations of experimental set-up and procedures. In **(A,B)** for illustrative purposes, the stimulus is depicted slightly to the right with respect to the subject's body. **(A)** Illustration of the baseline condition: the stimulus is positioned in front of the upright subject. **(B)** Illustration of the three experimental conditions when the subjects are lying on their left-ear side and the stimulus is positioned in front of them; the first disk from the left represents the above-head condition, the second disk represents the head condition and the last disk on the right indicates the below-head condition. **(C)** Picture showing the bar used to deliver the different stimulus orientations and the hand positions adopted by every subject during the exploration of the haptic stimulus.

In the baseline condition, subjects were standing up (see Figure 1A) whereas in the other conditions, subjects laid on their left side over a memory foam matrass and a pillow was added under their head in order to maintain head and body roll-tilted 90° counterclockwise relative to gravity (left ear down, see Figure 1B). In order to deliver the stimuli to be judged, we used a 3d printed white plastic bar (length: 1.5 cm; width: 1.2 cm; height: 17 cm) fixed over a black plastic circle (see Figure 1C). A section of 2 cm at one of the bar's ends had a texture rougher than the rest of the bar thus signaling the top. The bar was fixed on a computer controlled motorized arm. The position of the bar relative to the body changed in each condition, either at the shoulder level (baseline condition), above their head (above-head condition), in front of their head (head condition) or in front of their elbow (below-head condition) (see Figure 1B). Motor's sound potentially cueing bar's rotation was masked by white noise played over speakers for 2.5 s during stimulus rotation adjustment before stimulus exploration.

### Procedure

We ran a baseline and three experimental conditions. We used a 2 alternative forced choice task (2AFC) as this method has been found to be less vulnerable to artifacts compared to other methods as the adjustment task (Baccini et al., 2014). In the baseline condition, subjects were asked to verbally indicate whether the bar was tilted away from the horizontal either upward or downward; in the other conditions, we asked subjects to verbally indicate if the bar was tilted away from vertical either leftward or rightward; in order to avoid potential confusion given by the tilt on the roll plane, subjects indicated whether the bar was more tilted “toward the direction of their head” or “toward the direction of their feet.” In all conditions, subjects used their right hand to explore the bar (see Figure 1C). Sighted subjects were blindfolded in order to avoid visual information potentially influencing their response and the set-up was made not visible before and after each block of trials. Each experimental condition was run on a single block of 100 trials. Block order was pseudorandomized and presented over a period of maximum 4 days. All experiments were conducted in a darkened room.

### Psi method

**Figure 2:**
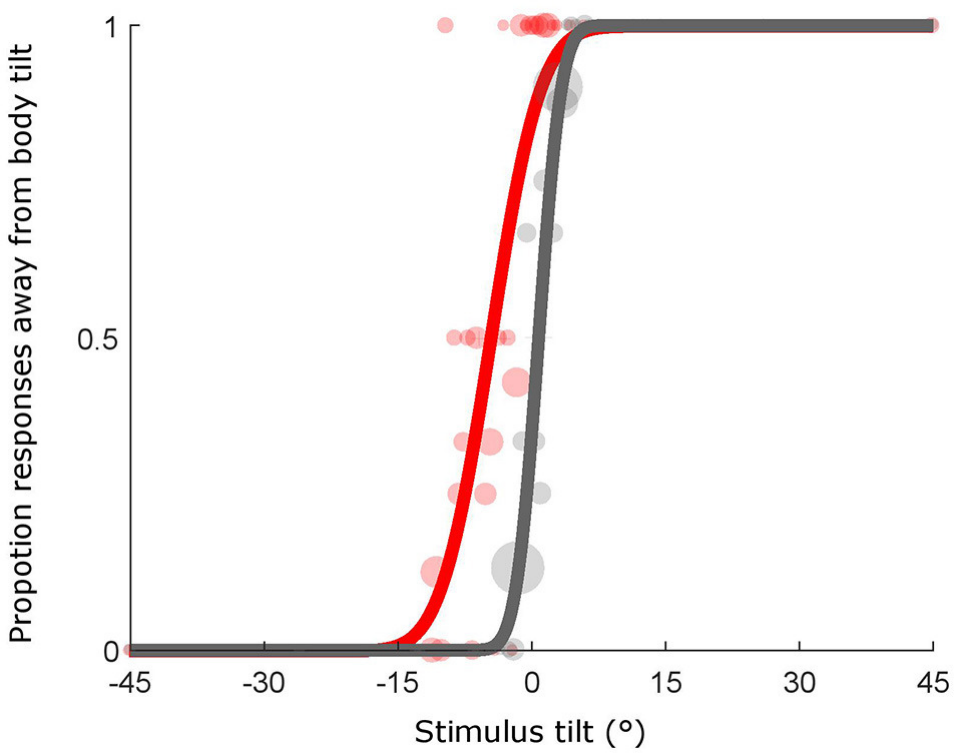
Example psychometric functions. Individual late blind subject data from the two conditions, baseline (gray line and shaded gray dots, goodness of fit: pDev = 0.85) and head (red line and shaded red dots, goodness of fit: pDev = 0.23) conditions are represented. Size of dots is proportional to the number of presentations for that particular stimulus orientation.

Orientation of the bar for each trial was determined by the Psi adaptive procedure (Kontsevich and Tyler, 1999), implemented using the PAL_AMPM routine from the Palamedes toolbox (Prins and Kingdom, 2009). This method takes advantage of a Bayesian criterion to minimize the uncertainty associated with the parameter estimates of the psychometric function (i.e., mean and SD of the cumulative Gaussian fit). For each condition and subject, we fit a cumulative Gaussian to the data using the PAL_PFML_Fit routine from the Palamedes toolbox (Prins and Kingdom, 2009) which finds the best fit in a maximum likelihood sense (see Figure 2). The mean provides a measure of the point of subjective equality (PSE) that is the orientation at which the bar is perceived to be vertical (in the experimental conditions) or horizontal (in the baseline condition). The sigma was extrapolated and used as a measure of precision associated with the estimate. The goodness of fit was calculated by means of the PAL_PFML_GoodnessOfFit function in MATLAB (Prins and Kingdom, 2009).

### Statistical analysis

In order to test whether there are inherent biases in the haptic readout of bar orientation we first compare the baseline PSEs across groups by means of two tailed one sample *t*-tests and paired *t*-tests corrected for multiple comparisons (Bonferroni correction for three comparisons). Results for the experimental conditions when subjects are tilted are analyzed by running a linear mixed model ANOVA to the PSEs, i.e., the perceived verticality in each experimental condition expressed in degrees, considering the experimental condition (head, above-head and below-head conditions) and the presence of blindness as factors. In order to test bias significance level, *post-hoc* analysis were conducted by means of two tailed one sample *t*-tests corrected for multiple comparisons (Bonferroni correction for three comparisons). Provided the heterogeneity of the visually impaired population, the analysis on this group was done by dividing subjects in subgroups depending on the onset of blindness (late vs. early visually impaired persons) or the presence of echolocation behavior (echolocators vs. non-echolocators). Therefore, a linear mixed model ANOVA was run considering experimental condition, blindness onset and echolocation behavior as factors. *Post-hoc* analysis are conducted by using two tailed one-sample *t*-tests corrected for multiple comparison (Bonferroni correction for three comparisons) focusing on the comparison between experimental conditions in each visually impaired subgroup as well as between subgroups for each experimental condition (i.e. late vs. early blind individuals and echolocators vs. non-echolocators).

## Results

The results for an individual late blind subject are depicted in Figure 2 showing the psychometric curve describing his performance in the baseline and the head conditions. The shift of the psychometric function is a measure of the bias in perceiving verticality and it is indicated by any deviations from 0 of the point of subjective equality (PSE). This value indicates the orientation in degrees at which the bar is perceived equally tilted to one side and the other. In the baseline conditions negative PSEs indicate downward biases and positive PSEs indicate upward biases while in the experimental conditions, positive PSEs refer to biases away from the body tilt and negative PSEs indicate biases toward the body tilt. For instance, in Figure 2 the individual subject shows a PSE of −4.4° in the head condition thus indicating a bias toward body tilt while the baseline is slightly biased upward (PSE = 0.7°).

One blind subject performance in the below-head condition and one sighted subject performance in the head condition were excluded from the analysis because their sigma values exceeded 4 SD from the mean of the sigma values of all participants.

**Figure 3:**
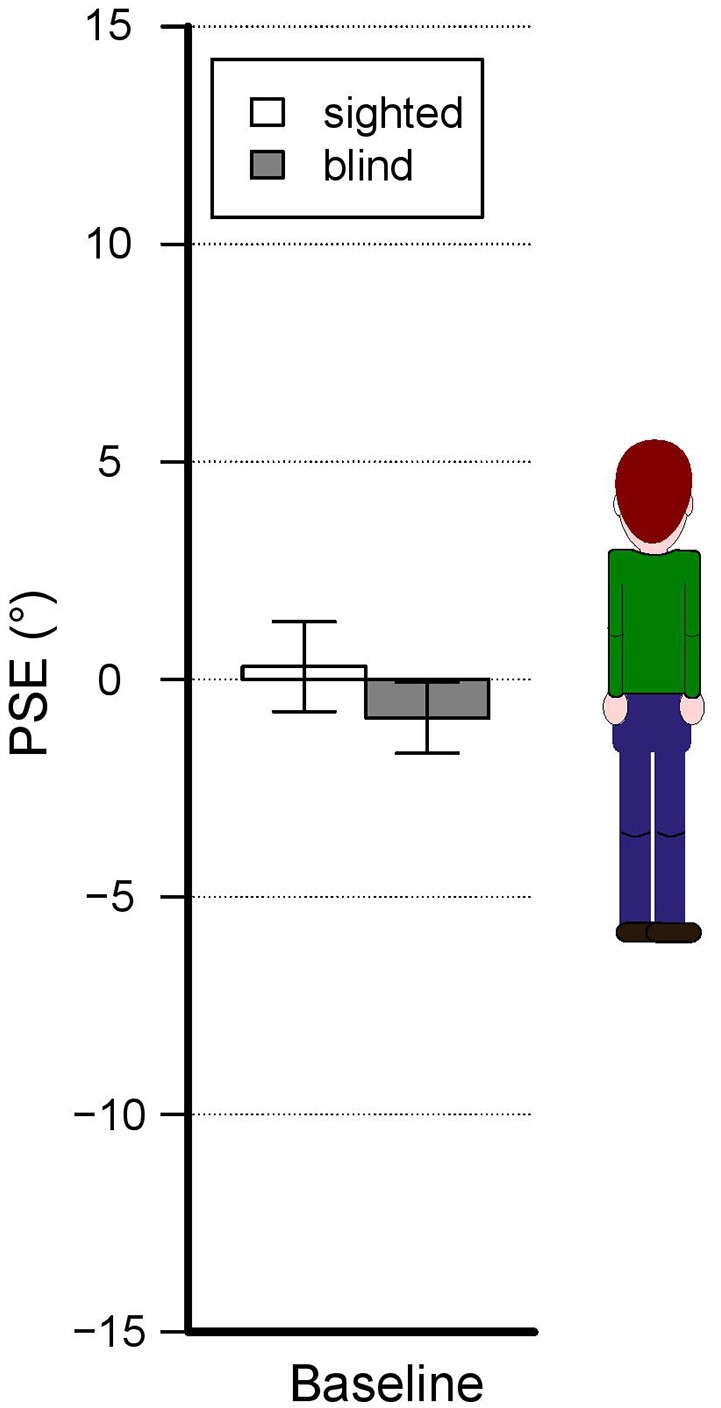
Baseline condition results—accuracy. The bars represent the mean PSEs across subjects. There is no significant shift relative to zero. Error bars show standard error.

**Figure 4:**
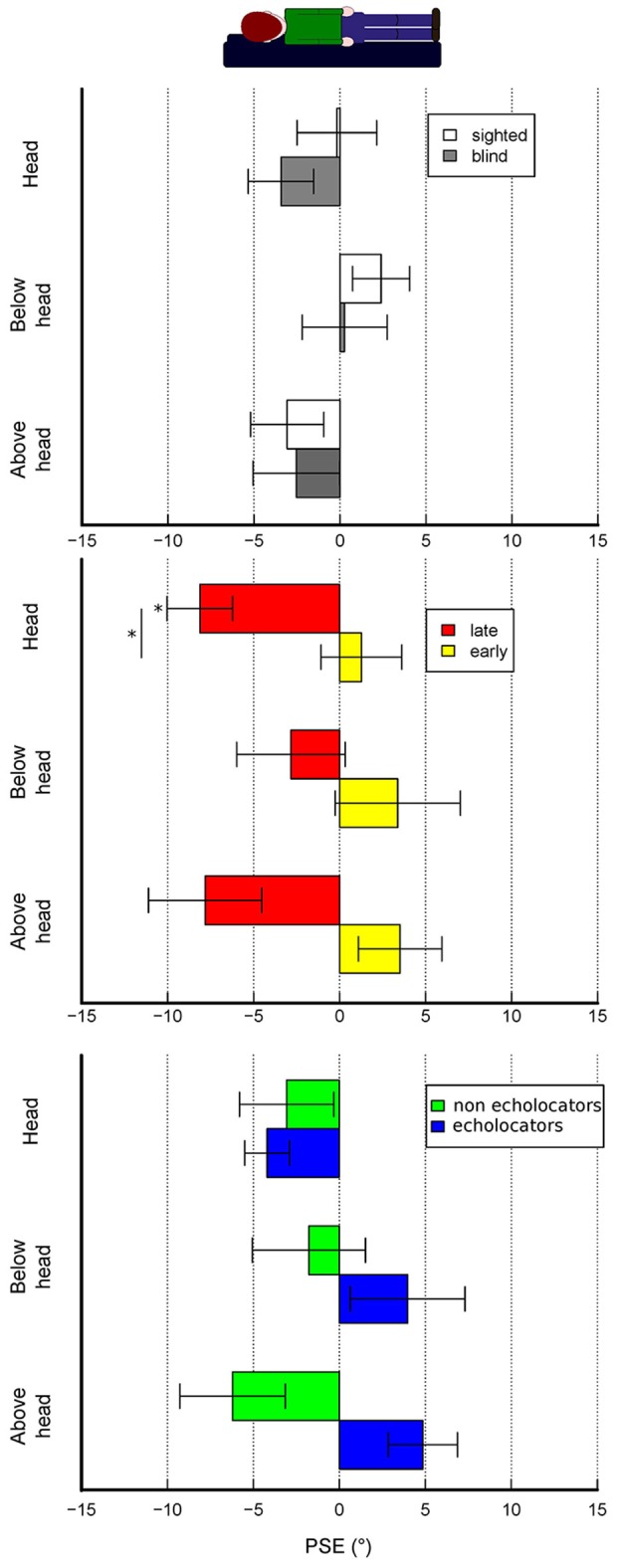
Experimental conditions results—accuracy. The bars represent the mean PSEs across subjects. Error bars show standard error. **(A)** Results for sighted and non-sighted participants are plotted depending on the three experimental conditions. Results regarding only visually impaired participants are plotted depending on subjects properties, namely either if they had early or late blindness onset **(B)** or if they engage in echolocation behavior during daily life **(C)**. Asterisks statistical significance (alpha = 0.05).

Estimates show different pattern of results depending on the presence of blindness, blindness onset and whether visually impaired subjects are echolocators or not. As shown in Figure 3, in the baseline condition subjects do not show significant biases regardless of the presence of blindness or not and also the comparison between the two groups show no significant differences. Generally, verticality estimates show biases that change depending on the experimental condition and the subject group (see Figure 4).

A linear mixed model ANOVA of PSEs that considers experimental condition and presence of blindness as factors shows that there is a significant effect given by the experimental condition [*F*_(2, 65)_ = 4.07, *p* = 0.02] but no effect given by blindness [*F*_(1, 44)_ = 0.31, *p* = 0.58] nor the interaction of the two factors [*F*_(2, 65)_ = 0.32, *p* = 0.72]. Nevertheless, blind population can differ depending on specific factors. Therefore, by focusing on this subject group we treated onset of blindness, either late or congenital, and presence of echolocation behavior during daily life as factors that could influence the behavioral results across all experimental conditions. We ran a linear mixed model ANOVA of PSEs showing a significant effect given by blindness onset either late or congenital [*F*_(1, 12)_ = 23.4, *p* < 0.001], a significant interaction of experimental condition with presence of echolocation behavior during daily life [*F*_(2, 21)_ = 5.2, *p* = 0.01], a significant interaction between blindness onset and echolocation habit [*F*_(1, 12)_ = 9.8, *p* < 0.01].

In all conditions, sighted participants show no consistent biases in any direction (mean PSEs ± SD, baseline: 0.29° ± 4.6, head: −0.18° ± 10.8, below-head: 2.39° ± 8.7, above-head: −3.06° ± 9.5; negative sign indicates a bias toward body tilt, positive sign a bias away from body tilt). Visually impaired subjects show no strong biases in all conditions (mean PSEs ± SD, baseline: −0.88° ± 3, below-head: 0.27° ± 9.2, head: −3.42° ± 7.5, above-head: −2.52° ± 9.7). In the abovementioned cases, *post-hoc* analysis did not show significant biases in the different experimental conditions. By focusing on the visually impaired subgroups we can observe a different pattern of results. Late blind subjects have significant biases toward the body tilt in the head condition (averaged PSEs, head: −8.12° ± 5.4, Bonferroni corrected *t*-test: *p* = 0.01) whereas no significant biases are observed in the other conditions. On the contrary, early blind subjects do not show significant biases in all conditions (mean PSEs ± SD for baseline = −0.20° ± 2; head = 1.27° ± 6.6; below-head = 3.38° ± 9.6; above-head = 3.51° ± 6.4). By focusing on the echolocation behavior, although the ANOVA reveals a significant interaction of echolocation behavior and experimental condition, *post-hoc* analysis did not reveal significant biases.

Paired *t*-tests reveal no significant differences between conditions in each group and visually impaired subgroup except for the echolocator subgroup where there is an almost significant difference between head and above-head condition (*p* = 0.06). Regarding the comparison between subgroups for each experimental condition we found a significant difference between early and late blind subjects in perceiving verticality at the head position (*p* = 0.02) and an almost significant difference when the stimulus is presented above the head (*p* = 0.05) whereas the other comparisons were not significant. Results regarding precision in perceived verticality are shown in the supplementary material.

## Discussion

In this study, we investigated haptic perception of verticality in sighted and visually impaired individuals when tilted counterclockwise. We tested whether there were inherent biases in judging bar's orientation when standing upright and we showed that estimates are not biased. When tilted counterclockwise we observed that biases change depending on several factors: blindness onset, position of the bar relative to the head on the body longitudinal axis and echolocation behavior during daily life.

Regarding sighted participants we only observe that the experimental condition has a significant influence on the perceived verticality but this was not revealed by the *post-hoc* analysis. It is possible that the brain accesses head and body coordinates when judging verticality at different positions on the longitudinal axis. Nevertheless, the absence of a significance level in the *post-hoc* analysis might rely on the high interindividual variability. Along these lines, previous studies investigating haptic perception of verticality when tilted on a side have found higher variability for the haptic rather than the visual perception of verticality when tilted on a side (see Introduction). Further investigation is therefore needed to better understand which individual properties might explain such variability.

Visually impaired subjects showed a different pattern of results. Concerning late blind subjects, we found consistent A-effects in the condition where the bar is aligned with the head position. Interestingly, we did not observe such a pattern of biases in sighted people. Moreover, when we consider early blind individuals, we observe no biases in perceiving verticality across all experimental conditions. As previously mentioned, haptic judgments of verticality have shown patterns of biases that tend to E-effects (Bauermeister et al., 1964; Schuler et al., 2010), A-effects (Bortolami et al., 2006; Fraser et al., 2015) or no biases at all when only the head is tilted (Tarnutzer et al., 2012), while visual judgments of verticality when roll-tilted show consistent biases toward head and body tilt. In this framework, the results presented here indicate a strong role of vision not only in gaining functional perception of object orientation (Gori et al., 2008) but also in influencing the haptic readout of verticality in interaction with the proprioceptive and vestibular sensory information signaling body roll tilt. This result suggests that the influence of priors does not develop in those individuals who did not experience vision during early stages of development. In this context, the development of an idiotropic vector signaling the most important posture humans need for achieving successful spatial navigation might be based on an ontogenetically generated visually defined prior. Such visual influence is stronger in the head reference frame as shown by verticality biases induced by head rather than body tilt in both the visual and the haptic modality (De Vrijer et al., 2009; Clemens et al., 2011; Fraser et al., 2015). The reason behind this difference might rely on a possible differentiation of vision and haptic systems after development. On the one hand, in comparison to early blind individuals, late blind subjects might anchor their visually acquired idiotropic vector to the haptic modality thus showing A-effects mostly when judgments are made in alignment with their head. On the other hand, in sighted individuals the haptic readout of perceived verticality might access body rather than head coordinates thus the bias gets reduced. In this sense, the presence of vision at the early stages of development may play a pivotal role in generating an idiotropic vector that is influencing also the haptic modality. Disambiguation of haptic and visual readout of verticality would appear in development but, in order to take place, vision might be needed especially at the head level where it is functional for building a reliable spatial representation of the surroundings. In other words, the presence of vision at early stages of development (as in late blind individuals) might allow for the formation of the prior: if vision does not get affected afterwards, disambiguation of visual and haptic readout of verticality may take place and this is reflected by less pronounced or absent biases in the haptic modality; on the contrary, when blindness is acquired after development the prior might remain or get anchored to the haptic modality as this is one of the senses used to explore space during spatial navigation, thus it shows A-effects. The absence of visual experience since birth would not allow for the formation of a prior for the upright position. Such reference would be useful in order to successfully navigate through space as it takes into account an internal reference of gravity that may help preventing the risk of falls. A visually gained prior could indeed be useful in conditions where a haptic feedback (e.g., by touching a wall next to us or a low ceiling) might need a stronger gravity reference and such task would require to process a larger portion of space thus extending from head to peripersonal coordinates.

Another possible characterization of the different verticality judgments observed in the visually impaired group might be related to their capability of spatially navigating through space. In order to investigate this aspect we focused on their echolocation habits. Echolocation is indeed used by many blind individuals and it has been shown to improve many aspects related to spatial navigation as auditory localization (for a review: Kolarik et al., 2014; Vercillo et al., 2015). Those blind subjects (3 early and 2 late) who reported to perform echolocation to orient themselves showed a pattern of biases that differs depending on the position of the haptic stimulus. When aligned with their head, verticality perception shows a tendency to A-effects whereas when presented above the head we observed a tendency to E-effects. This result suggests that a prior might be present at the head level only but not at the peripersonal space where perception of the surroundings might take advantage of other information acquired through the learned echolocation behavior. The reason behind this difference might rely on the fact that via echolocation, blind individuals improve their ability to move in space and in general their environmental spatial representation (Wallmeier and Wiegrebe, 2014; Kolarik et al., 2017). However, because of the absence of vision, blind individuals need to exploit the remaining sensory modalities and their navigational skills may be more dependent on the proprioceptive and vestibular sensory information rather than the other sensory modalities. In order to test this hypothesis, further studies are needed focusing on the spatial navigation capabilities and how these can be related to perception of gravity in the blind people population.

The study of verticality provides important insights on the role of vision not only in influencing other sensory modalities as haptic but also it allowed us to examine the influence of this factor in relationship to the proprioceptive and vestibular readout of body orientation in space. These aspects are of great importance to everyday tasks as spatial navigation and posture stabilization. Considering the blind clinical population, our findings may be integrated in the development of sensory substitution devices aiming to improve spatial awareness (Gori et al., 2016) and navigation through space (Cuturi et al., 2016) in order to increase and improve the functionality and utilization of scientific based rehabilitative devices.

## Author contributions

LC and MG conceived and designed the project. LC performed experiments. LC analyzed data. LC and MG wrote and edited the manuscript. All authors gave final approval for publication.

### Conflict of interest statement

The authors declare that the research was conducted in the absence of any commercial or financial relationships that could be construed as a potential conflict of interest. The reviewer RGG and handling Editor declared their shared affiliation.
