# Supplementary material for: The Effect of Visual Experience on Perceived Haptic Verticality When Tilted in the Roll Plane
Source: Front Neurosci. 2017 Dec 6;11:687. doi: 10.3389/fnins.2017.00687 (PMC5723665; doi:10.3389/fnins.2017.00687)
Supplement: Supplementary file 1 [file Presentation1.PDF]

Supplementary materials

Regarding precision (i.e. sigma obtained with the psychometric fit), a mixed model ANOVA show a significant effect given by experimental condition both when considering all subjects groups ($F(3, 97) = 30.38$, $p < 0.0001$) and also when focused on the visually impaired group ($F(3,31) = 9.60$, $p < 0.001$) and the sighted group ($F(3, 54) = 20.8$, $p < 0.0001$).

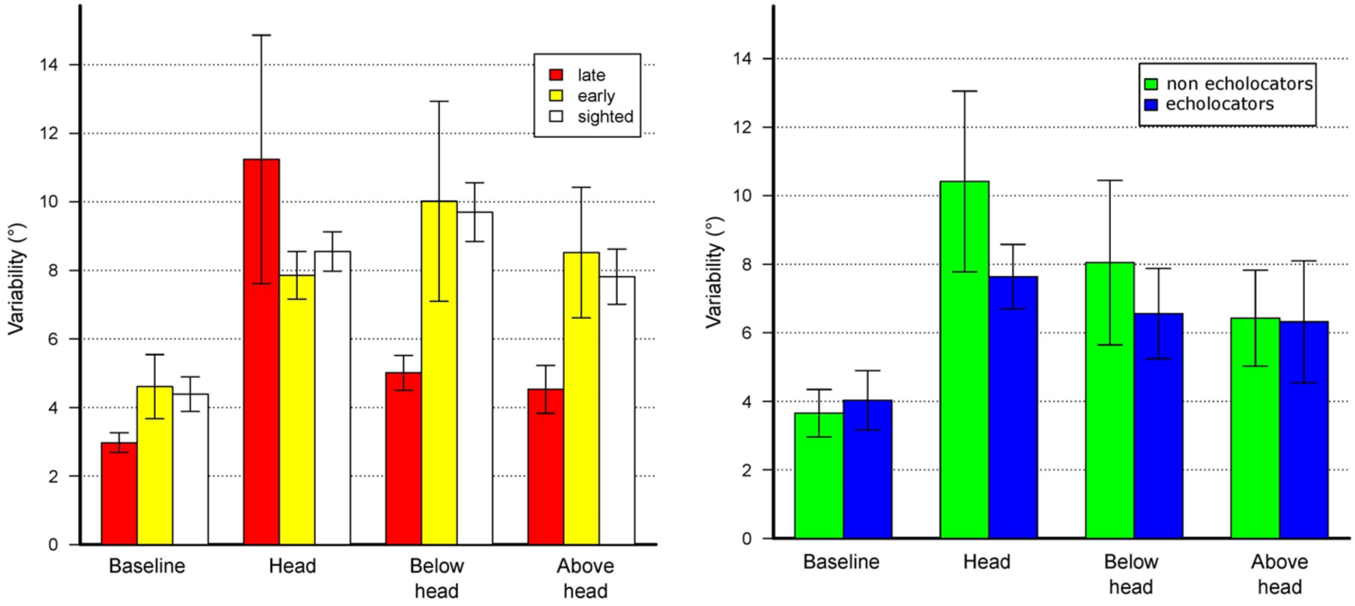

Figure S1. Precision results. Each bar represent the mean across subjects of the sigma taken from the psychometric fit to each individual subject data. Error bars show standard error. **A)** Averaged variability is shown for each condition in sighted and non-sighted subjects either with early or late blindness onset. **B)** Averaged variability for echolocator and non-echolocator participants in each tested condition.
